# Characterization of the Pancreatic Neuroendocrine Neoplasm Immune Microenvironment

**DOI:** 10.1002/cam4.70798

**Published:** 2025-03-27

**Authors:** Filipe Reis Neves, Ana Luís Martins, Rui Caetano Oliveira, Rui Martins

**Affiliations:** ^1^ Instituto Português de Oncologia de Coimbra Coimbra Portugal; ^2^ Centro de Medicina Laboratorial Germano de Sousa Coimbra Portugal; ^3^ Faculdade de Medicina da Universidade de Coimbra Coimbra Portugal

**Keywords:** immunotherapy, pancreatic cancer, tumor microenvironment

## Abstract

**Introduction:**

A tumor is composed of more than tumoral cells. In recent years, there has been an increase in interest and knowledge of the tumor microenvironment (TME).

**Methods:**

The TME is an integral part of the tumor, composed of several cells: immune, stromal, and endothelial, among others, thus offering a wide range of tumor interactions and multiple possibilities for targeted therapies and environment modulation. While the TME in pancreatic ductal adenocarcinoma is widely studied, it is not very true for the TME of pancreatic neuroendocrine neoplasms (PNENs).

**Discussion and Conclusion:**

The incidence of PNENs is increasing and, therefore, it is important to comprehend their biology for the evolution of efficient therapies since many of the PNENs develop metastasis, including the G1 PNENs. This paper focuses on a review of the role of the TME in PNENs.

## Graphical abstract

Enhancements in the creation of animal models that most accurately represent the aggressive types of pancreatic neuroendocrine neoplasms are necessary to create therapeutic protocols that combine immunotherapies with other medication classes to help patients with metastatic disease recover or respond to treatment more durably. Tumor microenvironment is thought to be essential to immunotherapy's effectiveness; nonetheless, assessing the immune microenvironment and its clinical ramifications remains difficult, particularly in neuroendocrine neoplasms.

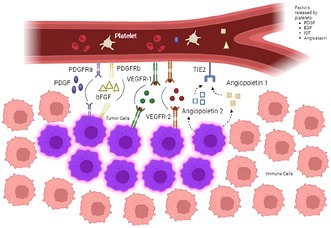

## 1 Introduction to Tumor Microenvironment (TME)

Analyzing a tumor's microenvironment offers insight into new treatment targets. Therapeutic options for gastroenteropancreatic neuroendocrine tumors (GEP‐NETs) are very limited, and at the same time, the incidence of these cancers is increasing [1].

We focus on pancreatic neuroendocrine neoplasms (PNENs) as they have a highly specific and complex tumor microenvironment. The incidence of neuroendocrine tumors has been increasing in the last decades, and currently, GEP‐NETs are the second most common digestive cancer in prevalence [2].

Studying PNENs microenvironment is clinically important due to the need for new therapeutic strategies, such as susceptibility to immunotherapy. The treatment modalities for advanced and well‐differentiated PNENs are rather limited, based on somatostatin analogs [3], and tumor regression is rare [4].

**FIGURE 1:**
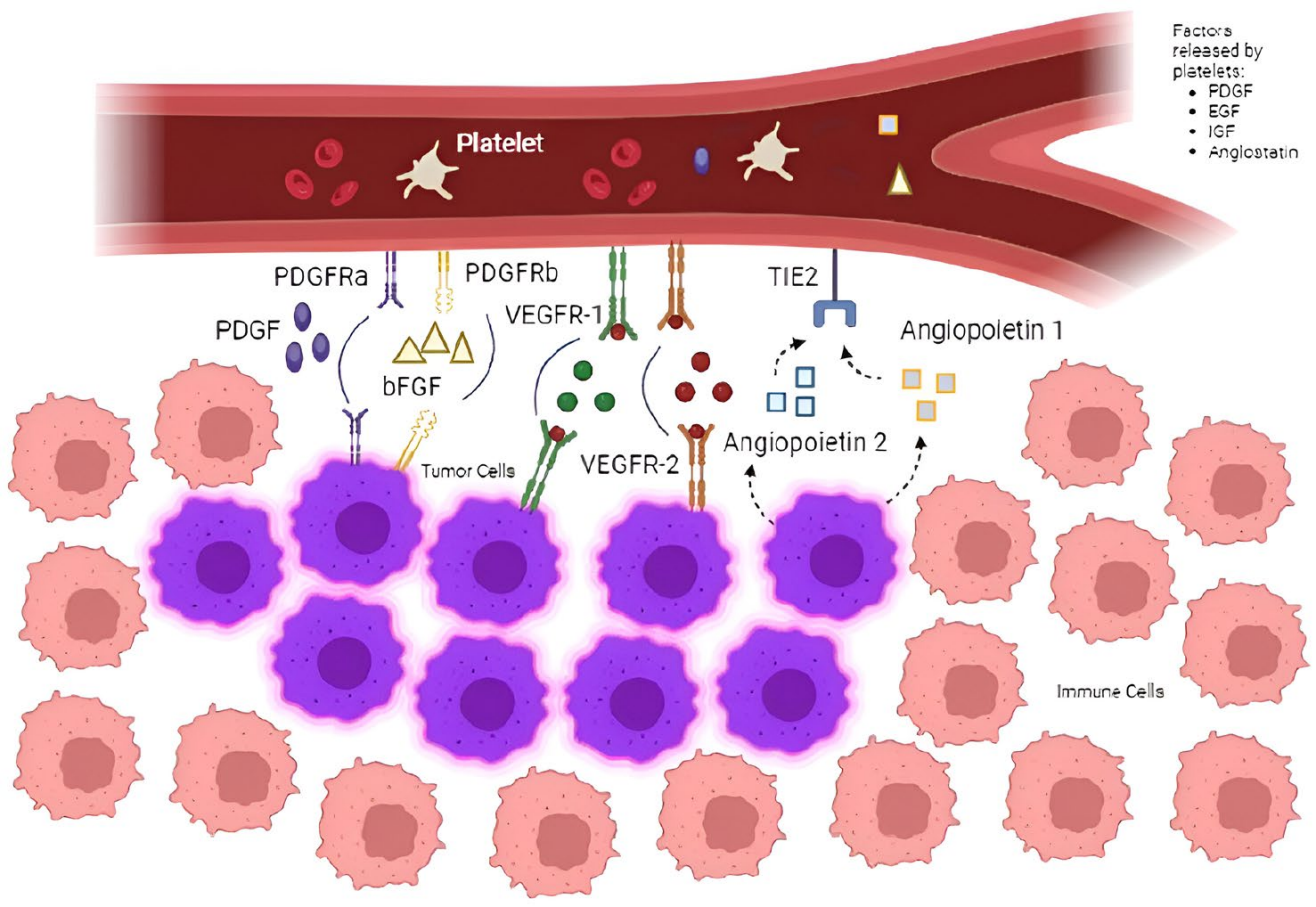
Schematic representation of angiogenesis in neuroendocrine tumors and the main molecular factors that regulate it (adapted from Yau, A.) [6].

The tumor microenvironment (TME) refers to the complex network of cells and structures that surround and interact with cancer cells within a tumor. This interaction is not merely structural but also functional, playing a direct role in the growth, proliferation, and spread of cancer cells [5]. Figure 1 shows a schematic representation of the angiogenesis molecular factors.

It is composed of tumor cells, immune system cells, stromal cells, and the interactions between them. The immune system serves as the first natural line of defense against cancer. The goal of tumor cells is to promote an immunosuppressive environment to evade the immune system and grow. Hence, we can envision a battle between two opposing immune responses [7].

The evaluation of tumor immune microenvironments is now a useful diagnostic tool for a variety of solid tumors due to recent developments in cancer immunotherapy, the fourth pillar of cancer treatment. The effective application of immune checkpoint inhibitors has recently marked a significant advancement in the field of cancer immunotherapy [8].

Extensive literature has investigated the host immune response against cancer and demonstrated the prognostic impact of immune infiltration in tumors. A methodology named the “Immunoscore” has been defined to quantify in situ immune infiltration. Notably, the infiltration of immune cells appears to be higher in PNENs, possibly because of the higher mutational burden of PNENs. Usually, studies obtain two RNA‐Seq datasets from PNEN patients and analyze the immune characteristics to determine the immune profile of this heterogeneous tumor, quantifying the expression of 10 immune markers (IL‐16, IRF4, LRG1, MUC1, CXCL9, CCL19, CR2, PIR, CD79A, and TCF21) via immunochemistry and CD4, CD8, and CD163 distribution intratumorally and peritumorally based on immunofluorescence [9].

Various biological parameters reflect systemic inflammation, such as elevated blood neutrophil‐to‐lymphocytes (NLR) ratio, which fosters tumor proliferation and metastasis via inhibition of apoptosis, promotion of angiogenesis, and DNA damage. Thus, the tumor‐associated neutrophils (TANs) were shown to interact with tumor cells. Depending on various extracellular stimulations (e.g., IFNg and TGF‐B), TANs may present an “immunosuppressive switch” from antitumor N1 phenotype to protumoral N2 phenotype. Moreover, TAN activity is dependent on the tumor type and location within the tumor (intratumoral versus stromal). Based on a cohort of 144 patients treated for localized or metastatic well‐differentiated PNEN, the NLR ≥ 4 was associated with worse overall survival [10].

There is evidence that the role of PIWI‐interacting RNAs (piRNA) in small extracellular vesicles (sEV) derived from PNENs plays a significant role in TME. PNEN‐derived sEV piR‐hsa‐30937 promotes CD276 expression in macrophages through the PTEN/AKT pathway and CD276þ macrophages suppress T‐cell antitumor immunity [11]. There is a summarized representation shown in Figure 1.

Targeting TME components or their signaling pathways presents a therapeutic intervention potential due to this dependence on the TME. Many efforts have been made to target TME components to gain therapeutic advantages in cancer patients, given the growing awareness of the critical functions of TME in tumor formation and therapeutic resistance [12].

There is a significant therapeutic advantage in targeting the TME compared with directly targeting cancer cells because cancer cells are prone to drug resistance due to their genomic instability, whereas nontumor cells in the TME have a genetically more stable nature and are more vulnerable [13].

As crucial elements of the immune system, macrophages are essential for the modulation of immunological responses, regulation of inflammatory processes, tissue remodeling, and surveillance of immune activities. Macrophages are activated when exposed to cytokines and various environmental factors, including microbial substances. M1 and M2 represent two distinct subtypes of activated macrophages. The M1 type signifies the phenotype that is classically active, while the M2 type signifies the phenotype that is alternatively activated. The M1 macrophages are essential for defense against intracellular infections and cancers. By producing tumoricidal agents such as interleukin (IL)‐12, IL‐6, and tumor necrosis factor‐α (TNF‐α), they remove detrimental tissue responses. In contrast, M2 macrophages inhibit the immune system and are commonly stimulated by IL‐4 and IL‐13. They produce cytokines such as transforming growth factor‐β (TGF‐β) and IL‐10 that initiate a T helper 2 (Th2) immune response. Higher levels of M2 macrophages correlate with larger tumors, early liver recurrence, local recurrence, and reduced survival time for pancreatic cancer patients [14].

The host's cellular antitumor response started against cancer cells via induction of cytotoxic T cells, natural killer cells, and monocytes/macrophages, whose action leads cancer cells to death. Therefore, the interaction between tumor cells and the host immune system via secretion of cytokines and soluble factors (or tumor‐derived supernatants) can contribute to cancer progression and, on the other hand, can affect each other's metabolisms and functions [15].

## 2 Cancer Therapies and TME in PNENs

Less than 3% of primary pancreatic neoplasms are PNENs; however, in recent decades, the frequency has increased. It is commonly recognized that PNENs exhibit a broad spectrum of clinical characteristics due to their extreme heterogeneity. Based on morphology (well differentiated vs. poorly differentiated), the Ki‐67 proliferation index, and/or the mitotic count, pancreatic neoplasms are categorized into Classes 1 through 3. Age and advanced‐stage or higher‐grade tumors are positively correlated with worse survival for patients with PNENs [16].

PNENs are categorized as either nonfunctional or functional according to whether tumor cells hypersecrete hormones. Functional tumors are defined as those that produce glucagon, somatostatin, insulin, gastrin, and vasoactive intestinal polypeptide (VIP) [17].

Given that PNENs are uncommon cancers that originate from the islets of Langerhans, their extremely complex tumor microenvironment plays a role in the tumor's resistance to anticancer treatments [18].

Ten percent of PNENs are caused by inherited tumor syndromes, such as Cowden syndrome, von Hippel–Lindau syndrome, multiple endocrine neoplasia type 1, multiple endocrine neoplasia type 4, neurofibromatosis type 1, tuberous sclerosis complex 1/2, Glucagon cell hyperplasia and neoplasia, and familial insulinomatosis [19].

Driver mutations in the MEN1, DAXX/ATRX, and mTOR pathway genes are linked to the initiation and growth of tumors in sporadic PNENs. In addition, there are germline mutations in MUTYH, CHEK2, BRCA2, PHLDA3, and the VEGF and Notch pathways, among other genetic abnormalities. Furthermore, alterations in the immunological microenvironment and microRNA were seen in PNEN [19].

Taking advantage of genomic or RNA sequencing, several recent studies considered the association of multiple genes in a global view and tried to connect these genes with clinical events, like metastasis or recurrence. Whole‐exome sequencing of 20 PNENs distant metastases, followed by a separate validation in 347 primary PNENs with/without distant metastases, found that 81% of metastatic PNENs cases involved loss or deletion in DAXX, ATRX, SETD2/H3K36me3, ARID1A, or CDKN2A. Cases that did not harbor any of these changes had a disease‐free 5‐year survival rate of 98% and a disease‐specific 10‐year survival rate of 95%. However, these survival rates dramatically decreased to 39% and 44%, respectively, for cases with any of the aforementioned mutations or with deletions [20].

Similarly, the resulting actions of the three protagonists have also been evaluated. In 64 well‐differentiated G1/G2 cases, 58% harbored the A‐D‐M mutant genotype (ATRX, DAXX, MEN1, MEN1/ATRX, and MEN1/DAXX mutations). The gene expression signature of the A‐D‐M mutant subtype (similar to α‐cell expression signature, which will be mentioned below) formed a tighter cluster and had smaller variance than A‐D‐M WT in hierarchical clustering and principal component analysis. As those with the A‐D‐M mutant genotype had worse recurrence‐free survival, this homogeneous subtype is expected to be associated with G1/G2 PNENs recurrence prediction [21].

**FIGURE 2:**
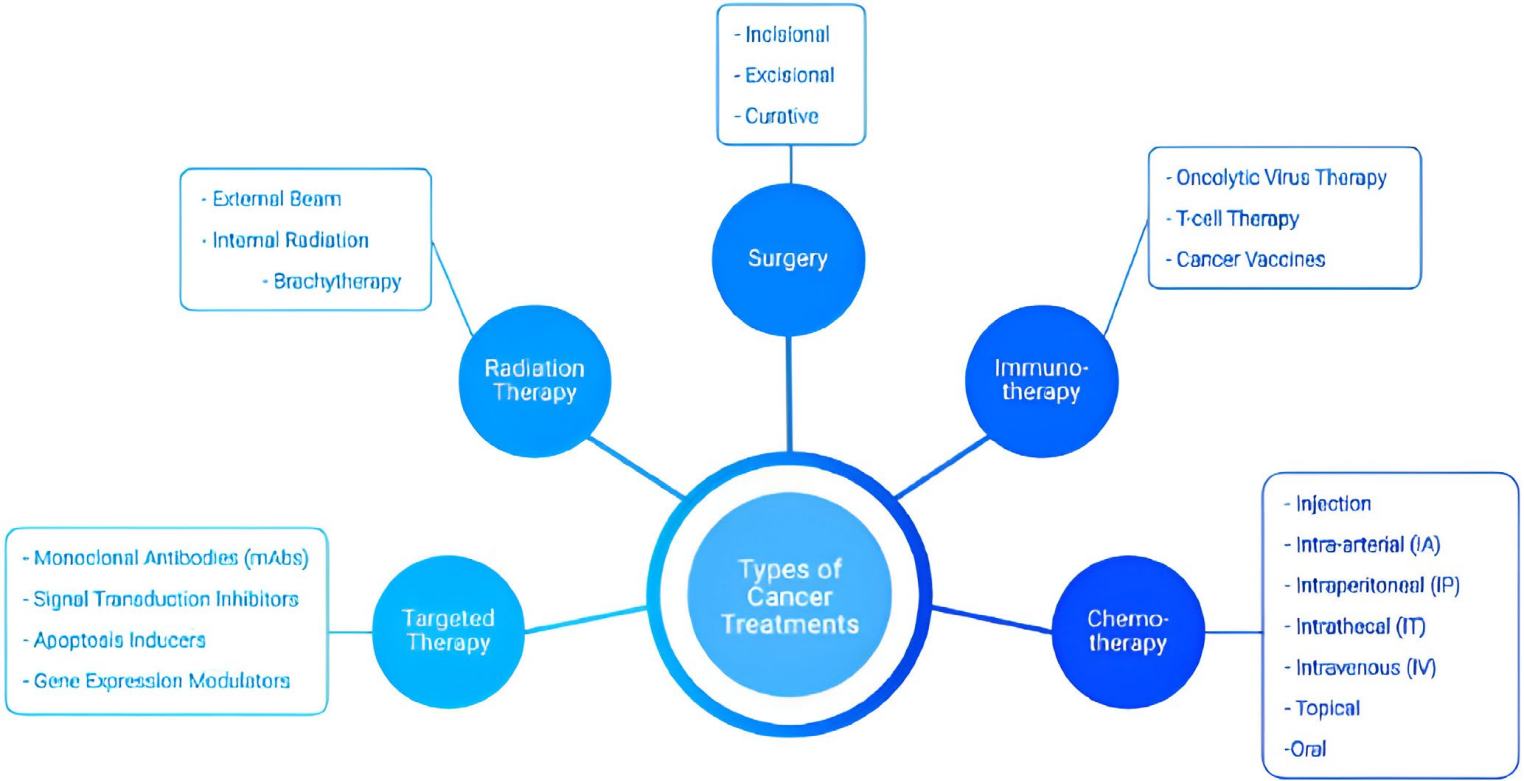
A few types of cancer treatment available for cancer patients (adapted from Xiao, Y.) [12].

Surgery is the treatment of choice for local or locoregional disease in PNENs G1 and G2. In functional GEP‐NETs, clinical symptoms should be managed before any intervention. Preoperative evaluation of localized Pan‐NETs should consider tumor size, the presence of unspecific symptoms, functional activity, localization of the lesion, and signs of local invasiveness. Several studies demonstrated the safety of a watch‐and‐wait strategy instead of surgery for asymptomatic PNENs ≤ 2 cm. Nevertheless, the shortness of follow‐up and the absence of prospective studies still suggest a cautious attitude towards this approach [22]. Figure 2 offers a schematic representation of therapeutic options.

A recent study by Zheng et al. investigated the immunological features of the TME in PNENs, aiming to identify prognostic biomarkers and explore the therapeutic potential of immunotherapy. Low expression of CD3, CD8, CD4, CD68, CD163, Foxp3, CD56, CD69, GZMB, HLA‐ I, HLA‐II, PD‐1, and PD‐L1 was observed in the majority of PNEN patient samples. PD‐1 expression was positively correlated with CD4 and Foxp3 expression, while PD‐L1 expression was positively correlated with CD68, CD163, and Foxp3 expression; HLA‐II expression was positively correlated with GZMB expression. Infiltration of lymphocytes (CD3+ or CD8+) and macrophages (CD68+ or CD163+) and expression of PD‐1/PD‐L1 were more pronounced in poorly differentiated neuroendocrine carcinoma compared to well‐differentiated neuroendocrine tumors, while CD68 and PD‐L1 correlated with advanced disease stage. The findings suggest that immunotherapy, particularly targeting the PD‐1/PD‐L1 pathway, may serve as a promising strategy for the treatment of PNENs, especially for poorly differentiated neuroendocrine carcinomas [23].

A recent study tried to immune type PNENs, assigning them into three clusters based on immune infiltration degrees. These clusters were termed as low immune cell infiltration subtype (Immunity‐L), middle immune cell infiltration subtype (Immunity‐M), and high immune cell infiltration subtype (Immunity‐H), respectively. To validate the above clustering result, tumor purity, immune score, and stromal score were calculated. It was found that high immune cell infiltration degrees were positively correlated with the stromal score and immune score, but tumor purity was the opposite. Besides, immune cell infiltration subtypes could predict metastasis [24].

## 3 Models for the PNENs Microenvironment

### 3.1 In Vitro Studies

Despite their drawbacks, in vitro experiments serve as valuable foundational models for translational research, offering a less complex evaluation of a particular route or the impact of potential drugs in human trials. There are not many cell lines used for PNENs; BON‐1, QGP‐1, and GOT‐1 are only a handful of the cell lines that have been employed in studies with reported findings. BON‐1 is the most popular kind of human cell line utilized in in vitro research. The cytogenetic changes in these cells are consistent with pancreatic neuroendocrine cancer [25].

The polyclonal BON1 cell line is one of the few human pancreatic neuroendocrine cell lines available to the research community and is thus widely used in endocrine tumor biology studies. Immortalized cells are derived from lymph node sporadic PNEN metastatic cells that produce menin, neurotensin, serotonin, and chromogranin A. There are limitations in using immortalized BON1 cells since their behavior is similar to that of neuroendocrine carcinoma G3. However, there is no available G1 human PNEN cell line. At least the BON1 cells are of pancreatic neuroendocrine origin and thus more relevant than other available human cell lines. It is known that long‐term culturing may result in genetic drift and phenotypic alterations of cell lines [26].

Besides human cell lines, animal cell lines provide different physiological and pathophysiological mechanisms to create in vitro models. The most used animal cell lines for PNEN studies are rat insulinoma cell lines, used to analyze tumor suppressor gene TP53 expression in PNENs. Besides these options, INS‐1832/13 rat insulinoma cell lines, mouse insulinoma 6 (MIN‐6) cell lines), and Cellosaurus beta‐TC‐3 (βTC3) cell lines are commonly employed, and SSTR2 is usually expressed in these cell lines [27].

### 3.2 In Vivo Studies

The C57BL/6, BALB/c, and FVB mouse strains are the most widely used models for immunotherapy preclinical investigations. In these models, tumor cells can be formed spontaneously, generated through carcinogens or transgenic manipulation. These cells are genetically related to the transplanted tumor cells, but they can be grown in vitro and employed as a source of inoculation for the host. The recipient mice are appropriate for trials involving immuno‐oncology therapy given that they possess a fully immunocompetent system [28].

The most popular model for studying the tumor microenvironment is the Rip1‐Tag2 model. The simian virus 40 big T‐antigen (Tag) oncogene is expressed under the rat insulin promoter (Rip) in these genetically modified mouse models, where it abrogates the Rb and p53 suppressor genes. In this system, the tumor resembles neuroendocrine carcinoma more than well‐differentiated PNENs and grows gradually through hyperplasia, dysplasia, adenoma, and carcinoma. At 10–12 weeks, tumors start to grow in Rip1‐Tag2 animals. Previous research indicates that they can exhibit invasive carcinoma in roughly 10% of cases [29]. They can be employed in preclinical research of antiangiogenic drugs and to analyze the tumor blood vascular network because they express VEGF‐A, an angiogenic molecule that is critical in tumor growth [30].

In addition to Rip1‐Tag2, another popular kind of murine model is the transgenic Rip1‐Tag5 mouse, which is widely employed in preclinical immune‐oncology research conducted by PNENs. Around 24 weeks, later than in the Rip1‐Tag2 model, tumor formation (insulinomas) occurs, and Tag is identified as nonself. This leads to an influx of autoreactive lymphocytes during the multistep tumor formation process [31].

In the Rip1‐Tag5 model, immune cell infiltration in the tumor microenvironment is recognized to be gradually lost during the tumorigenesis process, leading to solid tumors deprived of lymphocytes, in contrast to what is described in the human PNENs microenvironment, where immune cell infiltration increases according to tumor progression. A different study that also used this model revealed a decrease in the number of lymphocytes invading both the early stages of carcinogenesis and solid tumors [32].

Mice in the Rip1‐Tag2 line begin to express Tag during embryogenesis. The mice show systemic tolerance to Tag, mounting neither humoral nor cellular immune responses to Tag upon immunization with purified protein. Rip1‐Tag2 mice display a reproducible pattern of multistage tumor development, from normal islets to hyperplastic/dysplastic islets, to angiogenic islets, to varying grades of solid tumor, with a well‐defined angiogenic progression. All Rip1‐Tag2 mice succumb to hypoglycemia and tumor burden by 13–16 weeks. When Rip1‐Tag2 was crossed with TCR transgenic mice (TCR1) carrying Tag‐specific CD4+ T cells, temporal induction of T‐cell tolerance to Tag was detected in the pancreatic draining lymph nodes in the double transgenic mice. The T‐cell tolerance is manifested in reductions of both idiotypic CD4+ T‐cell abundance and of the T‐cell proliferation response to Tag in vitro [32].

Another line of Rip‐Tag mice, Rip1‐Tag5, has a different spatial and temporal order of Tag expression. Tag expression begins at approximately 10 weeks of age. Several weeks later, a spontaneous autoimmune response to Tag is readily detectable, with significant lymphocyte infiltration of hyperplastic/dysplastic islets. Mice in this line readily mount a humoral and CTL response to Tag, and as such, are nontolerant. Infiltration of premalignant lesions is variable and occasionally intense but is minimal in solid tumors. Crosses of Rip1‐Tag5 mice to transgenic mice that either increased the abundance of anti‐Tag CD4+ T cells or rendered the tumor cells costimulatory (Rip‐B7‐1) dramatically enhanced the infiltration of premalignant lesions, but not of solid tumors. Additionally, ex vivo stimulated Tag‐specific CD4+ T cells were transferred into Rip1‐Tag5. Like many of the adoptive transfers of tumor‐specific T cells in clinical cases, no significant lymphocyte infiltration into solid tumors was detected. If, however, Rip1‐Tag5 mice were treated with lethal radiation and subsequent reconstitution with bone marrow from RAG1‐deficient mice prior to T cell transfer, modest lymphocyte infiltration into solid tumors was noted [32].

### 3.3 Patients‐Derived Xenografts

PNEN patients often develop severe diseases that require systemic therapy. Patient‐derived xenograft models have been shown to be an effective tool for medication resistance, biomarker screening, preclinical trials, and drug development investigations [33].

Since PDX models maintain the gene expression and histological characteristics of the original transplanted tissue, such as the cancer stroma, they are regarded as beneficial models. However, these mice do not have a functioning immune system in microenvironment research. Human hematopoietic cells, human stem cell repopulation, and patient stromal tissue can be inserted into tumors to mimic tumor growth and microenvironment creation, which helps to partially address this difficulty [33].

Tumor growth inhibition was observed in studies utilizing humanized mice treated with anti‐PD‐1 antibody, recreated with cord blood‐derived CD34+ cells, and transplanted with tumor tissue. This led to an increase in tumor‐infiltrating IFN‐γ‐producing T cells and a decrease in regulatory T cells (Tregs) [34].

It is noteworthy to acknowledge that these models possess certain limitations as well. A limitation of PDX models is their limited longevity, which means that clinical scenarios like drug reduction or suspension cannot be replicated, making them inappropriate for long‐term studies. Furthermore, the ability to reproduce mice using autologous patient‐derived cells is not always feasible [24] (Table 1).

**TABLE 1:** Preclinical models for studying PNENs.

	Description	
In vitro studies	Human cell lines	BON‐1 is the most popular kind. The cytogenetic changes in these cells are consistent with pancreatic neuroendocrine cancer. When injected into nude mice, they create tumors that are histologically identical to the original PNENs.
	Animal cell lines	The most commonly used animal cell lines for PNENs studies are rat insulinoma cell lines, used to analyze tumor suppressor gene TP53 expression in PNENs.
In vivo studies	Rip1‐Tag2 model	The simian virus 40 big T‐antigen (Tag) oncogene is expressed under the rat insulin promoter (Rip) in these genetically modified mouse models, where it abrogates the Rb and p53 suppressor genes. In this system, the tumor resembles neuroendocrine carcinoma more than well‐differentiated PNENs and grows gradually through hyperplasia, dysplasia, adenoma, and carcinoma.
	Rip1‐Tag5 model	Around 24 weeks, tumor formation (insulinomas) occurs, and Tag is identified as nonself. This leads to an influx of autoreactive lymphocytes during the multistep tumor formation process.
Patient‐derived Xenografts	These models maintain the gene expression and histological characteristics of the original transplanted tissue, such as the cancer stroma. However, these mice do not have a functioning immune system in microenvironment research.	

## 4 Microenvironment Features

### 4.1 Tumor‐Infiltrating Cells

Only a modest amount of research has been done so far to determine how particular immune cells impact the onset and course of PNENs. A study evaluated the infiltration of immune cells in pancreatic neuroendocrine carcinomas with low‐, intermediate‐, and high‐grade pancreatic neuroendocrine tumors. Their findings showed that, in contrast to high‐grade tumors, which had many tumor‐infiltrating lymphocytes (TILs), PNENs displayed a “cold” immune milieu with few TILs. It is important to remember that distinct T cell populations are found in PNENs, indicating that T cells may play a critical role in either promoting or suppressing the advancement of PNENs [31].

Survival is impacted by variations in the immunological microenvironment that exist between PNENs and other NENs. PNENs had the lowest TIL infiltrates when compared to Neuroendocrine Carcinomas and Pancreatic Ductal Adenocarcinomas. Not only were there few CD4+ T cells overall in PNENs but there were also hardly any FoxP3+ T regulatory cells. A higher FoxP3+ score of 2 in the tumor was the only significant predictor linked to a lower survival rate, according to a study of 101 PNEN cases [35].

The presence of many T cell populations in peripheral nerve endometriomas, as well as studies showing higher levels of interleukin (IL)‐2, attest to the importance of T cells in neuroendocrine tumors. The primary source of IL‐2, which is essential for effector and memory T cell maintenance and development, is activated CD4+ and CD8+ T cells [36].

T‐cell lymphocytic infiltration has a particular impact on PNENs. When compared to small intestinal NENs, a study by Silva et al. found that T‐cell infiltrates were more abundant in PNENs; however, there was no difference in PD‐1 and PD‐L1 expression, which were low in both groups, and both cohorts showed high PD‐L2 expression [37]. This could be a helpful therapeutic strategy for NENs, and as PNEN tumors had a higher T‐cell infiltrate, these tumors might react more favorably. This is still to be established. When PNENs were compared to nonpancreatic NENs in the Bösch et al. study, PD‐1/PD‐L1 expression was more common in PNENs [38].

A study from 2018, conducted by Takahashi et al., gives some perspectives on this subject. They have shown remarkable differences between the PNEN and neuroendocrine carcinomas, as well as pancreatic ductal adenocarcinomas, with the latter being more immune active. The interesting finding regarding the PNENs is that using spatial segmentation, they were able to assess a correlation between PNEN grade (G1, G2, and G3) with TILs in the epithelium rather than in stroma) using CD3+/PD‐1 and CD204+/PD‐L1 characteristics. The findings suggest that PNEN G3 intensifies the immunosuppression when compared to PNEN G1/G2 and is different from NEC, which magnifies the immune response and subsequent immunosuppression, reinforcing the genetic different profiles between the entities and their distinct therapies [32].

Apart from the T cells detected in the microenvironment of PNENs, there is mounting data indicating the importance of macrophages in the formation and advancement of PNENs. Reduced CD68+ macrophage counts are associated with a much greater chance of surviving without illness. Furthermore, increased tumor‐associated macrophage infiltration was associated with a higher risk of recurrence in individuals with nonfunctional PNENs than in those with lower CD68+ macrophage scores [35].

A potential route of escape for programmed death receptor‐1 (PD‐1) and programmed death ligand 1 (PD‐L1)‐regulated immune response may include changes to these axes. For example, the grade of PNENs influences the amount of macrophage infiltration in relation to PD‐1 and PD‐L1 [39].

According to certain studies, only a small percentage of cases—between 3% and 17%—have been shown to express PD‐L1, while TILs have a high rate of PD‐1 expression—up to 23,5% [40]. In keeping with the high expression of PD‐L1, high TILs were linked to worse OS; they were also linked to greater grade and lymph node metastases. Additionally, these data support the G1/G2 PNENs' less immunologically active milieu, which restricts the available therapeutic options. The so‐called “cold tumor” does not exhibit the traits linked to a positive response to checkpoint inhibitors [41].

**FIGURE 3:**
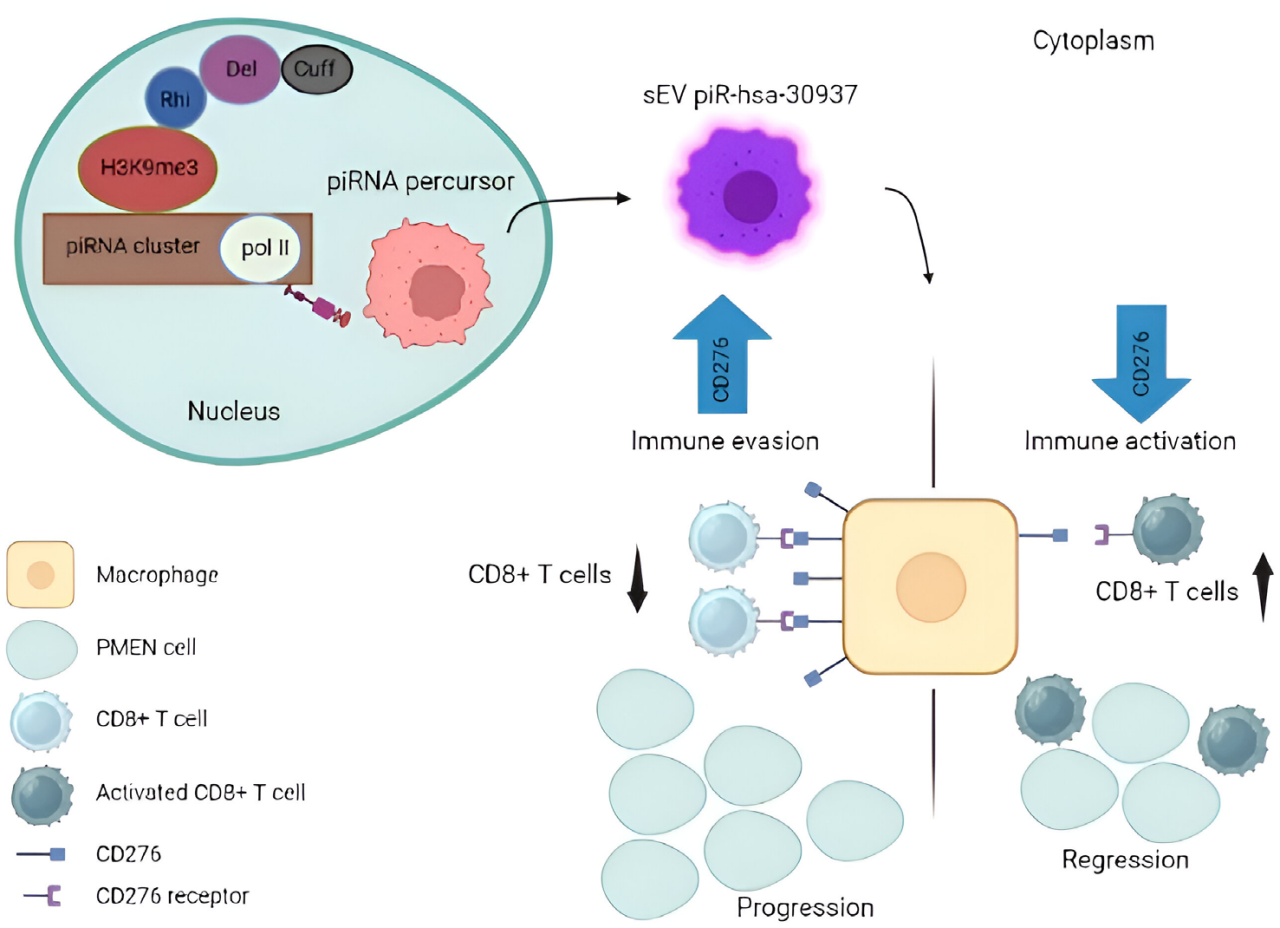
Schematic representation of the tumor immune microenvironment promoted by sEV piR‐hsa‐30937 in gastroenteropancreatic neuroendocrine tumors and the main factors that regulate it (adapted from Yao, AJ.) [47].

The loss or decrease in human leukocyte antigen (HLA) class I antigen presentation is another immunosuppressive mechanism that PNENs display. In fact, out of the 11 PNENs that were studied, it was found that 10 of them had changed expression of the HLA class I antigen. Additionally, it has been established that nearly every tumor taken from patients with PNENs had aberrant β‐2microglobulin expression. According to the study, the loss or reduction of HLA class I antigen in PNEN cells may alter how antigens are presented correctly, which may have an impact on how T‐cell–mediated immunity is modulated in PNENs [42]. The immune milieu has also been explored in the metastatic setting. The liver is the most common place of PNEN metastases, even after surgical resection [43] and the efficacy of adjuvant therapy is rather limited [44]. Some studies have explored the association between the number of TILs and postsurgical hepatic recurrence [45] but with some contradictory results [46]. A recent study from Greenberg et al. performed a very interesting and detailed analysis of the TME in metastatic PNENs. Resorting to several methodologies such as RNA‐Seq, flow cytometry, and Immunoscore, they found that PNENs infiltrating T cells had an activated phenotype, enriched in CD8+ T cells, and the ratio CD4/CD8 was different from the one in peripheral blood, supporting an immune response. Thus, metastatic PNENs were associated with a more robust immune microenvironment (low in localized PNENs) and exhibited an antigen‐experienced phenotype [18]. Figure 3 represents the importance of CD8+ T cells in the angiogenesis of tumors (Table 2).

**TABLE 2:** Particularities of tumor‐infiltrating cells in PNENs.

	Characterization
TILs	Lowest when compared to neuroendocrine carcinomas and pancreatic ductal adenocarcinomas
T‐cell infiltrates	More abundant when compared to small intestinal NENs
PD‐1/PD‐L1 expression	More common when compared to nonpancreatic NENs
HLA class I antigen	Out of the 11 PNENs that were studied, it was found that 10 of them had changed expression

### 4.2 Cytokines and Chemokines in PNENs

The endogenous cytokines that are generated during cancerous processes serve as either autocrine growth factors or markers of the tumor's immune response. The tumor microenvironment is shaped by chemokines and cytokines, which can either promote or inhibit tumor growth. The vascular endothelial growth factor (VEGF) family and its receptors are extensively expressed in PNENs, and it is well known that PNENs are highly angiogenic tumors [48].

Nonfunctional PNENs had greater serum levels of proinflammatory cytokines and chemokines, such as IL‐6 and IL‐8, as well as IL‐8 receptors, than functional PNENs. One important chemokine implicated in protumorigenic action is IL‐8, a strong neutrophil chemotactic agent. Increased IL‐8 overexpression resulted in increased cancer cell motility and proliferation, as well as an increased propensity for invasion. Furthermore, it has been shown that IL‐8 is a potent angiogenic factor and that elevated IL‐8 expression causes the angiogenic response to develop in vivo in a variety of tumor types [49].

Tumor‐induced HMGB1 has also been identified as a stimulator of cancer‐associated NETosis. The HMGB1/RAGE/IL‐8 axis plays a crucial role in neutrophil recruitment into the TME and NETosis as HMGB1 was found to bind the RAGE receptor on glioma cells, activating NF‐κB and upregulating IL‐8 expression—an influential neutrophil chemoattractant [50].

In a study using in vitro models of PNENs, additional cytokine analysis points to a connection between IL‐6 and C‐reactive protein (CRP). Specific immune cell types, such as macrophages or T cells, generate proinflammatory substances, including transforming growth factor‐β and IL‐6, which cause a rise in CRP, an acute‐phase protein [51].

CD40L‐CD40 interaction, one of the crucial interfaces between the innate and adaptive responses, plays a decisive role in modulating cytokines and consequential effector T cells in pathogenic responses, germinal center formation, affinity maturation, survival, proliferation, memory, autoimmunity, cancer, transplantation, and atherosclerosis. CD40L binding of CD40 results in multiple signaling events that lead to pleiotropic functions. The receptor CD40 lacks the kinase domain; it recruits TNF‐α receptor associated factors (TRAFs) at the cytoplasmic domain upon activation [52].

A different investigation on C‐X‐C motif chemokine ligand (CXCL) 12, also referred to as stromal cell‐derived factor‐1α, showed that in patients with PNENs, greater serum levels of this chemokine were linked to worse overall survival (OS), progression‐free survival (PFS), and poor treatment response [53].

### 4.3 The Extracellular Matrix (ECM) in PNENs

The ECM is a key component of tissue, both normal and tumoral, and it is composed of an intricate network of proteins with functions in structure, elasticity, cell migration, and tissue remodulation, among other functions [54].

In the neoplastic process, the ECM experiences alterations due to multiple signaling effects. This process is more evident in carcinomas [55], especially in PDAC [56]. In PNENs, the ECM is not so well characterized, but some changes have been described using mouse models, gene expression analysis, and immunohistochemistry [57]. PNENs can have upregulation of tenascin, heparanase, periostin, and fibrinogen‐α/β/γ [58], with downregulation of trypsin, trypsinogen, and lectin, among others [59].

The extension of ECM remodeling has been associated with grade, with higher‐grade usually associated with higher tissue modification. These dynamic changes in the EMC are usually attributed to two major families of β proteins—heparanases and matrix metalloproteinases [60].

Very interestingly, there is a difference in the proteins that are up‐ or downregulated, according to the NEN location [61] which may be helpful in the clinical setting; these proteins may even be present in serum or urine, prompting additional biomarkers for detection [62].

In EMC, a major component is fibroblasts. Their contribution to cancer development and progression has rapidly expanded. There is evidence of a cross‐talk not only between fibroblasts and PNEN cells but also between fibroblasts and inflammatory cells. This interaction is regulated by the PNEN cells through the secretion of several soluble factors such as serotonin, TGF‐β, and PDGF [63].

In cancer, fibroblasts acquire a specific phenotype—the cancer‐associated fibroblasts (CAFs). This phenomenon is well described in PDAC [64] but in PNENs, this is still poorly understood. In vitro studies have demonstrated that CAFs stimulate NET growth and that this interaction is affected by tumor grade [65], with less‐differentiated tumors having a minor dependence on this mechanism for proliferation [66].

NETs are highly vascularized tumors, especially when compared to carcinomas; well‐differentiated tumors are more vascularized than less‐differentiated lesions, and this has a good correlation with outcome and survival [67]. This has been explained by the capacity of the well‐differentiated lesions to promote a dense vascular network, whereas, in poorly differentiated lesions, the main mechanism is driven by hypoxia and neoangiogenesis [68]. This results in a microenvironment with overexpression of VEGF [69]—up to 80%—thus one of the main drivers of tumorigenesis [70].

## 5 Treatment and Targeting the PNENs' TME With Immune‐Modulating Agents

It can be difficult to treat both nonfunctional and functional PNENs. Currently, individuals with PNENs have only one curative goal option: surgery [13]. Furthermore, demonstrating the potential of immunological checkpoints in conjunction with antiangiogenic medications is the TME's use as a tumor drug target for diagnostic value, prognosis prediction, and efficacy evaluation [71].

### 5.1 Immunotherapy

Evidence has demonstrated that immune checkpoint inhibitors, such as PD‐1/PD‐L1 inhibitors, are effective in treating a variety of neoplasms. The best course of action for treating GEP‐NETs remains undetermined due to the multitude of potential origins, sites, and tumor characteristics [1]. Targeting PD‐L1 and its receptor, immune checkpoint‐based therapy has demonstrated efficacy against several malignancies recently, but not yet against well‐differentiated PNENs.

Using PD‐1 and PD‐L1 inhibitors may be a viable treatment option for patients with PNENs, as the expression of these two proteins is thought to be a possible prognostic marker for PNENs [72]. The fact that in an in vivo model of PNENs, anti‐PD‐L1 therapy sensitized tumors to antiangiogenic therapy is particularly intriguing. This study found that anti‐PD‐L1 treatment boosted the sustained efficacy of antiangiogenic therapy, particularly when it created intratumoral high endothelial venules that aided in a larger T cell infiltration and the death of tumor cells [73].

Immune checkpoint inhibitors have been promising in cancer, but well‐differentiated NETs do not seem to be an optimal candidate due to the lack of PD‐1/PD‐L1 expression in vivo; also, most NETs are mismatch repair proficient and have a low tumor mutational burden (TMB); the opposite is seen in NEC, so they seem the adequate candidate for this option [60].

### 5.2 Targeted Therapy

Targeted agents have also broken into the therapeutic landscape of patients with PNENs. Considering the expression of vascular endothelial growth factor receptors (VEGFR), platelet‐derived growth factor receptor (PDGFR) α and β, and stem cell factor receptor (c‐kit) in PNENs, antiangiogenic agents such as sunitinib, pazopanib, cabozantinib, or lenvatinib are or have been under research in this group of patients, showing considerable antitumoral activity [74].

The high vascularization of PNENs has given birth to many antiangiogenic treatments, but in most cases, only the VEGFR inhibitor sunitinib has demonstrated efficacy, and it is approved for the treatment of PNENs [75]. According to the CABINET trial, sunitinib will soon be replaced by cabozantinib [76]. Sunitinib has also been tested in association with evofosfamide (Phase II trial SUNEVO NCT02402062), exploring the synergistic activity, especially under hypoxia conditions (more evident in advanced and progressive PNENs, due to the deranged vasculature) with results coming out soon [77].

In a tentative effort to explore the microenvironment, namely, the mast cells and macrophages infiltrating PNENs, some interesting results emerged from animal models—ibrutinib demonstrated the capacity to suppress the degranulation of mast cells, resulting in vascular collapse and tumor regression [78]. Currently, there is an ongoing Phase II study based on this scenario—NCT02575300.

### 5.3 Immune Response‐Based Therapy

Immune response‐based therapy may be essential for preventing the progression of PNENs. Therapy targeting immune cells and/or cytokines in PNENs is still not well understood. Nonetheless, certain research indicates that strategies impacting immune response reprogramming may have a part in the development and manifestation of symptoms in PNEN patients. Research has shown that individuals with metastatic gastroenteropancreatic cancers respond similarly to interferon (IFN)‐α and somatostatin analog therapies in terms of antiproliferative effects [79].

Recently, there has been major interest shown in regulatory T cells (Tregs) and the tryptophan‐degrading enzymes indoleamine 2,3‐dioxygenase (IDO) and tryptophan 2,3‐dioxygenase (TDO). These enzymes convert tryptophan into kynurenine along the kynurenine pathway, which could lead to depletion of tryptophan in the tumor microenvironment. Tryptophan is also the precursor of serotonin. IDO and TDO are especially of interest in PNENs, as these tumors often produce serotonin, which potentially depletes its precursor, tryptophan [80]. Clinical trials based in this feature have been developed, with or without PD‐1 inhibitors [81]. The elevated levels of Tregs in PNENs may stem from the heightened expression of IDO and TDO by tumor cells, as well as CAFs, indicating that a combined strategy may be required [82].

This is in line with findings that CD4+ FoxP3+ Treg cells are described as independent predictors of poor prognosis due to their ability to work as main agents of immune evasion in cancer [45].

### 5.4 TNF Inhibitors

These observations suggest that low doses of TNF‐α vessel remodeling and immune response against PNEN progression, and therapy based on TNF‐α seems to be a useful approach as an immunotherapy for patients with PNENs [16].

NK cells have also been an object of study in PNENs. Their cytolytic activity seems to be diminished and associated with disease status, with variation in activeness according to disease progression or response to therapies [83].

Tumor‐associated macrophages (TAMs) arouse interest in NENs. They have been found in high amounts in poorly differentiated tumors and liver or lymph node metastases [84]. The number of TAMs in PNENs was correlated with outcomes and liver dissemination, mainly due to an angiogenic switch [85] (Table 3).

**TABLE 3:** Treatment options and ongoing clinical trials for patients with PNENs.

	Characterization		
Surgery	Only curative goal option.		
PD‐1/PD‐L1 inhibitors	Targeting PD‐L1 and its receptor, immune checkpoint‐based therapy has demonstrated efficacy against several malignancies recently, but not yet against well‐differentiated PNENs.		
Antiangiogenic agents	Only the VEGFR inhibitor sunitinib has demonstrated efficacy, and it is approved for the treatment of PNENs.		
	According to the CABINET trial, sunitinib will soon be replaced by cabozantinib.	Sunitinib has also been tested in association with evofosfamide (Phase II trial SUNEVO NCT02402062).	Ibrutinib demonstrated capacity to suppress the degranulation of mast cells, resulting in vascular collapse and tumor regression Currently, there is an ongoing Phase II study based on this scenario—NCT02575300.
Immune response‐based therapy	Therapy based on TNF‐α seems to be useful approach as an immunotherapy for patients with PNENs		
	Evidence of Tregs was detected by immunohistochemistry in PNENs, especially if they had serotonin production. Clinical trial bases in this feature have been developed, with or without PD‐1 inhibitors (Phase III trial OPTiM).		
TNF inhibitors	Therapy based on TNF‐α seems to be useful approach as an immunotherapy for patients with PNENs		

## 6 Summary and Perspective

The clinical outcomes of immunotherapy have been astonishing, but also disappointing. The diverse nature of these tumors presents an additional layer of hurdles for PNENs translational research. For PNENs to advance, clinical and translational research must be combined. Enhancements in the creation of animal models that most accurately represent the aggressive types of PNENs are necessary to create therapeutic protocols that combine immunotherapies with other medication classes to help patients with metastatic disease recover or respond to treatment more durably [16].

All things considered, within the past 10 years, research on NENs has advanced. For advanced malignancies, new treatment approaches, such as immunotherapy, have been introduced. Immunocheckpoint inhibitor therapy can also result in markedly higher response rates and, in certain situations, even long‐lasting, full responses. Even though immunotherapy has been shown to be successful in numerous clinical trials, there is still disagreement over the ideal patient population for this treatment. TME is thought to be essential to immunotherapy's effectiveness; nonetheless, assessing the immune microenvironment and its clinical ramifications remains difficult, particularly in NENs [1].

## Author Contributions

**Filipe Reis Neves:** conceptualization (equal), data curation (equal), resources (equal), software (equal), writing – original draft (equal), writing – review and editing (equal). **Ana Luís Martins:** conceptualization (equal), data curation (equal), resources (equal), validation (equal), visualization (equal), writing – original draft (equal), writing – review and editing (equal). **Rui Caetano Oliveira:** conceptualization (equal), data curation (equal), formal analysis (equal), investigation (equal), methodology (equal), project administration (equal), resources (supporting), supervision (supporting), validation (supporting), visualization (supporting), writing – review and editing (supporting). **Rui Martins:** conceptualization (equal), data curation (equal), formal analysis (equal), resources (supporting), supervision (supporting), validation (supporting), visualization (supporting), writing – review and editing (supporting).

## Conflicts of Interest

The authors declare no conflicts of interest.

## Data Availability

The data that support the findings of this study are openly available in PubMed at https://pubmed.ncbi.nlm.nih.gov/.
